# Effects of pretreatment strategies on fertility outcomes in patients with adenomyosis

**DOI:** 10.3389/frph.2024.1484202

**Published:** 2024-12-09

**Authors:** Gaby Moawad, Youssef Youssef, Arrigo Fruscalzo, Slim Khedhri, Hani Faysal, Paul Pirtea, Benedetta Guani, Alexandre Vallée, Jean Marc Ayoubi, Anis Feki

**Affiliations:** ^1^ Department of Obstetrics and Gynaecology, The George Washington University Hospital, Washington, DC, United States; ^2^ Divison of Minimally Invasive Gynecology, Department of Obstetrics and Gynecology, Maimonides Medical Center, Brooklyn, NY, United States; ^3^ Department of Obstetrics and Gynaecology, Fribourg University Hospital, Fribourg, Switzerland; ^4^ Department of Obstetrics and Gynaecology, Indiana University, Indianapolis, IN, United States; ^5^ Department of Obstetrics and Gynaecology and Reproductive Medicine, Hopital Foch–Faculté de Médecine Paris, Suresnes, France

**Keywords:** adenomyosis, pregnancy, infertility, *in vitro* fertilisation, gonadotropin-releasing hormone agonist, levonorgestrel intrauterine system, surgery, high-intensity focused ultrasound

## Abstract

Adenomyosis is a commonly encountered pathology in women of reproductive age and frequently coexists with infertility. The effect of adenomyosis on fertility, particularly on *in vitro* fertilisation and intracytoplasmic sperm injection outcomes, is not well understood. Various pretreatment modalities have been used to improve pregnancy rates and live birth outcomes; however, because of a lack of high-quality evidence, there is no clear consensus on the best pretreatment option. This review was conducted through a PubMed search aiming to highlight the relationship between pretreatment and fertility in women with adenomyosis. Medical, ablative surgical, and non-surgical therapies were reviewed. According to the current literature, gonadotropin-releasing hormone agonist therapy and placement of a levonorgestrel intrauterine system are two suitable medical pretreatment strategies that can improve the clinical pregnancy rates of patients with adenomyosis. Surgical ablation of adenomyosis can also be beneficial, although surgical management can be challenging. Non-surgical thermal techniques, including high-intensity focused ultrasound ablation, percutaneous microwave ablation, and radiofrequency ablation, are much less invasive techniques that have shown effectiveness in improving fertility. Although evidence remains limited, all these procedures have demonstrated a favourable safety profile. Further studies are needed to better develop these techniques and demonstrate their effectiveness.

## 1 Introduction

Adenomyosis is a benign disorder histologically defined by the presence of heterotopic endometrial glands and stroma within the myometrium (1). Advances in non-invasive diagnostic imaging modalities such as two- and three-dimensional transvaginal ultrasound and magnetic resonance imaging have increased the diagnostic accuracy without the need for histopathologic examination of excised tissues (2–4). The estimated incidence of adenomyosis is approximately 1%, which is equivalent to 29 per 10,000 person-years (5). Multiple theories have been proposed to explain its aetiology, including Müllerian rests, metaplasia of stem cells, genetic mutations, and endometrial invagination into the myometrium; however, the exact aetiology has not been determined (1, 6–8). The clinical presentation of adenomyosis usually involves dysmenorrhoea, menorrhagia, or abnormal uterine bleeding (9). Women of reproductive age with adenomyosis also experience poor obstetrical outcomes, such as miscarriages, preterm delivery, preterm rupture of membranes, and infertility, placing a considerable physical and psychological burden on these patients (10–13). Adenomyosis appears to negatively affect the outcomes of *in vitro* fertilisation (IVF) and intracytoplasmic sperm injection (ICSI) as evidenced by reduced clinical pregnancy and implantation rates (14, 15).

Multiple factors play a role in the reduced fertility rates associated with adenomyosis. These include inflammatory mediators such as tumour necrosis factor-α and interleukin-1 as well as free oxygen radicals induced by ectopic lesions (16–18), which are potentially toxic to embryos and disrupt endometrial receptivity (19–21). Thickening of the subendometrial junctional zone, a classic feature of adenomyosis, is also associated with significantly lower IVF implantation rates (22).

The optimal treatment choice for adenomyosis-related infertility remains unclear (23, 24). Fertility-sparing surgeries are indicated for focal lesions, but these procedures are complex and not always feasible (25). Gonadotropin-releasing hormone agonist (GnRHa) therapy has been proven to induce a hypoestrogenic effect and reduce tissue inflammation (26). GnRHa agents exert a direct anti-proliferative effect on the myometrium by centrally downregulating gonadotropin secretion; they also induce apoptosis in adenomyotic tissues (27). GnRHa therapy has even been proven to improve fertility in mice with induced adenomyosis by restoring endometrial receptivity (28). Levonorgestrel-releasing intrauterine system (LNG-IUS) has also been associated with downregulation of oestrogen receptors and alteration of steroid-metabolising enzyme pattern (29–31). Indeed, an extensive decidualisation of endometrial stromal cells and atrophy of the glandular and surface epithelium can be appreciated as early as 4 weeks after local LNG-IUS exposure (32).

Cytoreductive surgery may be another treatment option when more conservative therapy has failed. The first surgical treatment of extensive adenomyosis was reported by Osada et al., who proposed an open surgical technique for removal of diffuse disease with a complex triple-flap technique for uterine reconstruction (33). Several other techniques have since been proposed for treatment of both focal and diffuse adenomyosis, and a minimally invasive approach is feasible with some of these methods (34). Nonetheless, data regarding clinical fertility outcomes remain limited.

Non-surgical ablative techniques can be considered in selected patients for whom medical treatments have failed and fertility improvement is desired. Several technologies for thermal ablation of adenomyotic lesions or diffuse adenomyosis have been proposed, including high-intensity focused ultrasound (HIFU) ablation, percutaneous microwave (PMW) ablation, and radiofrequency (RF) ablation (35). Research on these techniques has greatly improved, showing promising fertility results; however, data are still limited.

No clear treatment plan has been established to address adenomyosis-related infertility. Therefore, this review was performed to explore the effectiveness of non-surgical pretreatment with GnRHa therapy or LNG-IUS placement in patients with adenomyosis undergoing IVF/ICSI. We also review the efficacy of surgical and non-surgical ablative treatment options, such as HIFU, RF ablation and PMA ablation techniques.

## 2 Literature search

For this narrative review, we searched PubMed using the terms “adenomyosis”, “*in vitro* fertilisation”, and “pretreatment” to identify the relationship between pretreatment and fertility outcomes in women with adenomyosis, particularly those undergoing IVF/ICSI. More in-depth search strategies were then implemented for the different pretreatment modalities, combining “adenomyosis” with the following MESH terms: “fertility”, “infertility”, “pregnancy”, “pretreatment”, “gonadotropin-releasing hormone agonist”, “progesterone intrauterine device”, “levonorgestrel-releasing intrauterine system”, “combined oral contraceptives”, “*in vitro* fertilisation”, “intracytoplasmic sperm injection”, “surgery”, “cytoreduction”, “ablation”, “high-intensity focused ultrasound”, “percutaneous microwave”, and radiofrequency’. The search was limited to articles in English. Abstracts were screened to select relevant studies. The inclusion criteria were randomised controlled trials, case–control studies, cohort studies, case series, case reports, and systematic reviews and meta-analyses. The exclusion criteria were publication in any language other than English, letters to the editor, and video articles.

The SANRA (scale for the quality assessment of narrative review articles) criteria were applied when performing the literature research (36). Accordingly, they included the following six items: an explanation of (1) the importance and (2) the aims of the review, (3) the literature search and (4) refer-encing and presentation of (5) the evidence level, and (6) relevant endpoint data (36).

## 3 Impact and physiopathology of adenomyosis-related infertility

Adenomyosis is a common issue in sub-fertile and infertile women. According to a recent meta-analysis of 21 longitudinal studies involving 25,600 women, the overall pooled prevalence of isolated adenomyosis was found to be as high as 10%. Adenomyosis was frequently found to coexist with other gynaecological pathologies, such as endometriosis [6%; 95% confidence interval (CI), 3%–11%], fibroids (1%; 95% CI, 0%–4%), or both (7%; 95% CI, 2%–13%) (37).

Notably, adenomyosis negatively affects fertility at different levels. It has been shown to reduce the pregnancy rate (both spontaneous and after IVF), the rate of ongoing pregnancy, and the live birth rate. A recent meta-analysis on this topic showed that compared to women without adenomyosis, those with adenomyosis had an increased risk of miscarriage [odds ratio (OR), 3.40; 95% CI, 1.41–8.65] (13) and lower rates of live birth (OR, 0.59; 95% CI, 0.37–0.92), clinical pregnancy (OR, 0.66; 95% CI, 0.48–0.90), and ongoing pregnancy (OR, 0.43; 95% CI, 0.21–0.88) (38).

In addition, like endometriosis, adenomyosis enhances the risk of obstetrical complications. These include an increased risk of premature birth (OR, 3.09; 95% CI, 1.88–5.09), small-for-gestational-age newborns (OR, 3.23; 95% CI, 1.71–6.09) (39), pre-eclampsia (OR, 4.32; 95% CI, 1.68–11.09), and post-partum haemorrhage (OR, 2.90; 95% CI, 1.39–6.05) (40).

The type of adenomyosis (focal vs. diffuse and internal vs. external) does not appear to influence the fertility outcome (40, 41). By contrast, the extent of adenomyosis, considered to be the total volume calculated by the sum of the volume of each lesion, appears to be a relevant prognostic factor for the reproductive outcome (42).

The mechanisms underlying infertility in adenomyosis are still unclear. Multiple factors are likely to be involved, with different pathways overlapping each other. This is especially likely in patients with coexisting pathologies such as endometriosis or myomas, tubal patency, ovulatory and endocrine dysfunctions, altered endometrial receptivity, and age-related infertility (43).

Several mechanisms of action have been proposed. In one mechanism, adenomyosis disrupts the physiological architecture of the junctional zone and myometrium, leading to dyssynergic uterine hypercontractility and fibrosis that negatively affect sperm and embryo transport (44). Recent literature emphasizes the impact of abnormal uterine contractility and retrograde uterine menstruation in the pathogenesis of endometriosis. An increased contraction frequency and amplitude have been, not only during menstruation, but across other phases of menstrual cycle seem to be responsible for the typical signs and symptoms of endometriosis, including fertility issue (45). Similarly, an abnormal pattern of uterine contractility was highlighted in patients affected by adenomyosis, suggesting a potential linking to infertility and dysmenorrhea (46).

Also, the development of progesterone resistance with the loss of progesterone paracrine signalling in the endometrium seem to have a negative impact in fertility (47). Another proposed mechanism is based on the inflammatory environment, which increases intrauterine oxidative stress. This leads to reduced endometrial receptivity to blastocyst attachment and implantation (48). Finally, inflammation can also result in imbalanced production of adhesion molecules and decreased expression of factors that seem to be crucial for implantation, such as HOXA10 and leukaemia inhibitory factor (49).

## 4 Pretreatment strategies for adenomyosis-associated infertility

**Figure 1:**
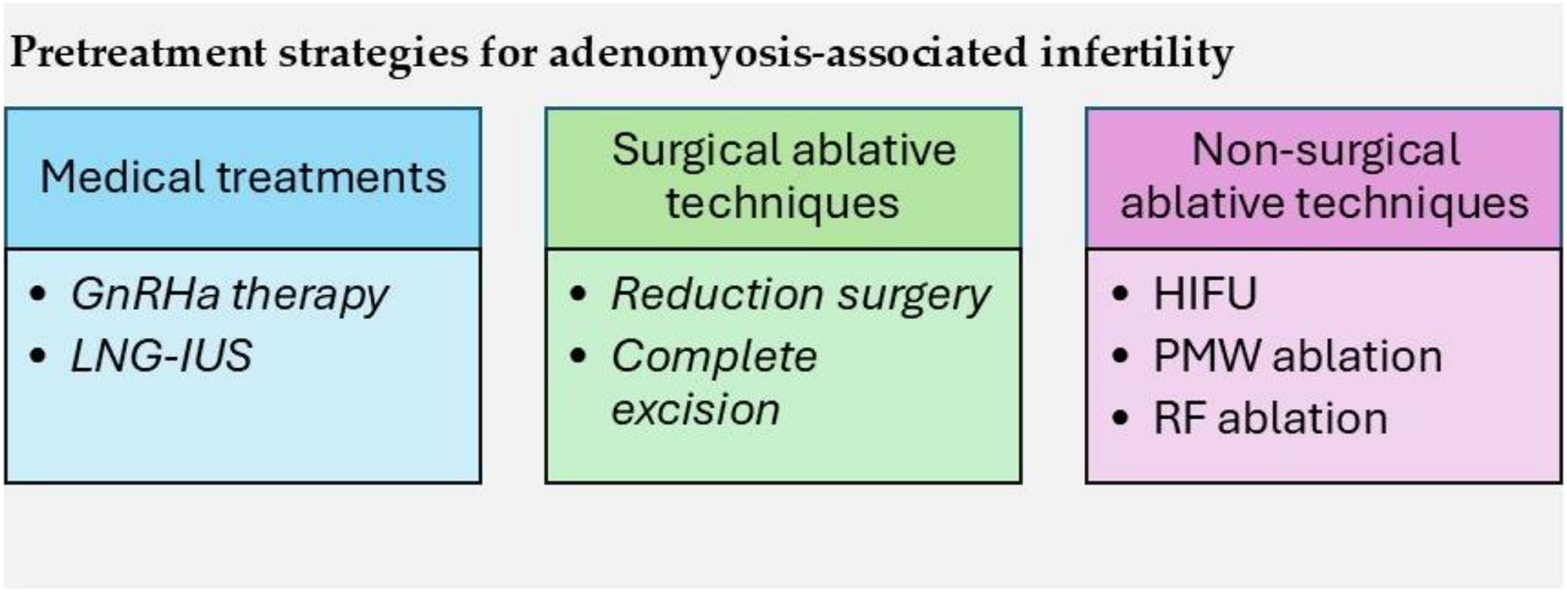
Pretreatment strategies for adenomyosis-associated infertility. GnRH, gonadotropin-releasing hormone agonist; LNG-IUS, levonorgestrel intrauterine system; HIFU, high-intensity focused ultrasound; PMW, percutaneous microwave; RF, radiofrequency.

In this section, we explore the different treatment options for adenomyosis-associated infertility. Strategies are presented and discussed in terms of fertility outcomes, particularly in women undergoing IVF and ICSI therapies. These strategies include medical treatments as well as surgical and non-surgical ablative techniques (Figure 1).

### 4.1 Medical treatments

#### 4.1.1 GnRHa therapy

GnRHa agents exert a direct anti-proliferative effect within the myometrium by acting on GnRH receptors expressed by adenomyotic lesions; they also induce a systemic and local hypo-oestrogenic effect (38). Moreover, GnRHa agents have been demonstrated to cause apoptosis in adenomyotic tissues and reduce inflammation and angiogenesis (27). A meta-analysis also suggested that GnRHa therapy improved the clinical pregnancy rates of patients with endometriosis (50).

Wu et al. (10) attempted to evaluate whether long-term GnRHa pretreatment before frozen embryo transfer improved pregnancy outcomes in women with adenomyosis. Three groups were compared: Group A underwent frozen embryo transfer cycles following long-term GnRHa pretreatment, Group B underwent fresh embryo transfer with the ultra-long GnRHa protocol, and Group C received fresh embryo transfer but with long GnRHa pretreatment. Patients assigned to the ultra-long GnRHa protocol received GnRHa for at least 3 months before starting ovarian stimulation (10).

Significantly higher implantation and live birth rates were found in Group A than in Groups B and C (implantation: 43.5% vs. 36.5%, and 43.5% vs. 30.8%, respectively; live birth: 50.9% vs. 40.9%, and 50.9% vs. 33.9%, respectively) (10). The authors also found that long-term GnRHa pretreatment and frozen embryo transfer were significantly protective factors for the implantation rate (A vs. C: OR, 1.729), clinical pregnancy rate (A vs. C: OR, 1.665), live birth rate (A vs. C: OR, 1.694), and miscarriage rate (A vs. C: OR, 0.203). In fresh embryo transfer cycles, ultra-long GnRHa pretreatment was significantly protective for the live birth rate (B vs. C: OR, 1.792) and miscarriage rate (B vs. C: OR, 0.333) (10). The authors concluded that long-term GnRHa pretreatment tended to improve the pregnancy rate for women with adenomyosis (10).

Aksenenko et al. (51) evaluated women with infertility and ineffective IVF attempts who were subsequently diagnosed with various stages of adenomyosis. The patients were divided into three groups: those who received treatment with GnRHa agents, combined oral contraceptives, or dienogest continuously for 3 months (51). Regardless of the type of preparatory therapy, the IVF outcomes were comparable to those of no treatment in patients with stage 1 adenomyosis (51). The treatment groups had significantly better pregnancy rates (by 5%–8%) in women with stage 2 adenomyosis than in the comparison group; however, no statistically significant differences were observed between the different therapy groups (51). GnRHa pretreatment increased the pregnancy rate by 5%–12% per IVF attempt for women with stage 3 adenomyosis, whereas pretreatment with combined oral contraceptives and dienogest had no significant therapeutic effect (51). However, the authors noted that the pregnancy rate remained low in patients with stage 3 adenomyosis, and they defined stage 3 adenomyosis as a cause of severe uterine infertility (51).

Two comparative studies evaluated the effects of GnRHa pretreatment before IVF cycles in women with adenomyosis. The first study by Niu et al. (52) compared the combination of GnRHa with add-back therapy vs. add-back therapy only before frozen embryo transfer. The second study by Park et al. (53) evaluated GnRHa pretreatment vs. no treatment before fresh embryo transfer. The results showed that GnRHa pretreatment appeared to be beneficial to the pregnancy rate (14). However, the authors noted that only two studies were available for their analysis at the time (14).

Li et al. (54) performed a retrospective analysis of 341 patients with adenomyosis undergoing frozen embryo transfer after IVF. The study group underwent GnRHa downregulation treatment based on the hormonal replacement therapy cycle, while the control group was treated solely with hormone replacement therapy (54). Interestingly, the authors found no statistically significant differences between the two groups with regard to the clinical pregnancy rates (40.63% vs. 42.54%, *p* = 0.72) or live birth rates (23.75% vs. 23.75%, *p* = 0.74) (54). However, they claimed that the cycles of GnRHa administered could havave been not sufficiently effective in patients with adenomyosis and also that results were probably negatively influenced by the inclusion in the study of patients without severe adenomyosis (54). Nonetheless, they acknowledged the possibility that GnRHa treatment might be ineffective (54).

Chen et al. (55) hypothesised that the supraphysiological hormone levels induced by controlled ovarian hyperstimulation may negate the benefits of GnRHa therapy during IVF. In their retrospective single-centre cohort study, all 374 patients received the long GnRHa protocol, with the study group additionally receiving GnRHa pretreatment (55). Interestingly, the live birth rate in the group that did not receive pretreatment was significantly higher than that in the GnRHa pretreatment group (37.7% vs. 21.2%, respectively; *p* = 0.028). The clinical pregnancy, miscarriage, and preterm labour rates were also not significantly different (55). The authors concluded that GnRHa pretreatment before the long agonist protocol did not improve the live birth rate in fresh embryo transfer (55).

Cozzolino et al. (38) performed a systematic review and meta-analysis focusing on the effect of adenomyosis on the clinical outcomes of IVF and ICSI in addition to the effects of GnRHa therapy and surgical treatments. They included the three studies by Niu et al. (33), Park et al. (34), and Chen et al. (36) and compared the effects of previous GnRHa therapy with no previous intervention. Notably, they found that pretreatment with GnRHa agents alone did not improve the clinical pregnancy rate (*p* = 0.55), disagreeing with the previous meta-analysis by Younes and Tulandi (14, 38). Two other studies included in their meta-analysis evaluated the outcomes of natural conception, considering the cumulative pregnancy rate 3 years after GnRHa pretreatment combined with surgical excision vs. GnRHa pretreatment alone (38, 56, 57). GnRHa pretreatment alone, without conservative surgery, was found to be a protective factor for the live birth rate (OR, 0.15; 95% CI, 0.05–0.51; *p* = 0.002), meaning that conservative surgery and GnRHa therapy also increased live birth rates (38). Additionally, no statistically significant difference was observed in the miscarriage rate between women receiving combined therapy and those receiving only GnRHa treatment (*p* = 0.99) (38). The authors stated that while pretreatment with GnRHa did not show a beneficial effect on IVF outcomes, conservative surgery with GnRHa seemed to restore fertility in women with adenomyosis (38). However, they concluded that they could not rule out the possibility that long-term GnRHa treatment and the long protocol might have a therapeutic effect on adenomyosis (38).

#### 4.1.2 LNG-IUS

The LNG-IUS, although originally designed for contraception, has also been introduced as a treatment for adenomyosis (58). It works by releasing 20 μg/day of levonorgestrel into the uterine cavity, with concentrations in the endometrium being 100-fold higher than those after administration of oral progesterone (59). The LNG-IUS has been proven to benefit women with endometriosis and adenomyosis via symptomatic control (58, 60–63).

Liang et al. (64) performed a retrospective study to evaluate the effect of pretreatment with an LNG-IUS on IVF and vitrified-warmed embryo transfer outcomes in women with adenomyosis. They included 358 women with adenomyosis undergoing IVF, assigning 134 to the LNG-IUS group and 224 to the control group (64). The ongoing pregnancy rate per transfer was significantly higher in the LNG-IUS group than in the control group (41.8% vs. 29.5%, respectively; *p* = 0.017) (64). Both the implantation rate (32.1% vs. 22.1%, *p* = 0.005) and the clinical pregnancy rate (44.0% vs. 33.5%, *p* = 0.045) were also significantly higher in the LNG-IUS group (64). Moreover, logistic regression analysis showed that the ongoing pregnancy rate was significantly associated with LNG-IUS use (adjusted OR, 1.628; 95% CI, 1.011–2.622). Overall, the authors demonstrated that pretreatment with an LNG-IUS prior to frozen embryo transfer improved the ongoing pregnancy rate, clinical pregnancy rate, and implantation rate for patients with adenomyosis undergoing IVF. Further randomised controlled trials using different study designs are needed to better evaluate the effect of LNG-IUS placement on IVF outcomes in women with adenomyosis (64).

### 4.2 Surgical ablative techniques

Cytoreductive surgery has been proposed for partial or complete removal of adenomyotic lesions. Reducing the amount of adenomyosis or, ideally, completely eliminating adenomyosis has been thought to restore the uterine anatomy and its underlying complex physiology. Nonetheless, such surgery can be technically challenging, the results can be incomplete, and complications can occur. Particularly problematic are obstetrical complications resulting from a scarred uterus (65).

Various surgical techniques have been described, including both laparoscopic minimally invasive approaches and open surgery (34). In analogy to the well-known surgical techniques used for myomectomy, adenomyotic tissue can be removed with a scalpel or by using electrosurgery and diathermy. The uterine wall is then sutured in multiple layers, often using different closing techniques, to restore the thickness of the myometrium. Overlapping techniques may be used to repair large uterine wall defects, suturing the remaining seromuscular flap into double or triple layers (66).

Relatively few studies have evaluated the fertility outcome after surgery for adenomyosis (67). The pregnancy rates after surgical treatment vary widely among the different studies that have been performed to date. In the largest study published, 30% of 70 women who desired a pregnancy became pregnant. Of the 70 women, 49 had undergone IVF therapy and 21 had achieved a natural pregnancy, with a birth rate of 76%, miscarriage rate of 19%, and stillbirth rate of 6% (68). Another retrospective study reviewed the fertility outcomes of 102 women after laparoscopic adenomyomectomy (69). Overall, conservative surgery improved the fertility of women who had experienced IVF treatment failure, particularly those younger than 39 years of age. The clinical pregnancy rate was 31.4%; however, this was largely impacted by maternal age, reaching 41.3% in patients younger than 39 years but only 3.7% in those older than 39 years (69). Another study evaluated the cumulative 3-year fertility outcome in patients undergoing conservative surgery vs. medical therapy by GnRHa. The results showed significantly higher clinical pregnancy and successful delivery rates in the surgery than medical therapy group (46.4% vs. 10.8% and 32.1% vs. 8.1%, respectively) (70).

Even fewer data are available on the efficacy of surgery followed by medical therapy. A retrospective study of nine patients who underwent excision of adenomyosis followed by a 6-month course of GnRHa therapy showed an increase in the pregnancy rate (one-third of the patients become pregnant) (71). Wang at al. compared the outcomes at the end of a 2-year follow-up period of women treated with conservative surgery with or without GnRHa therapy (72). The authors found a lower symptom relapse rate in the combined surgical–medical treatment group. However, they found no differences in the reproductive outcomes between the two groups (72).

When considering surgical ablation of adenomyosis, clinicians must keep in mind the risk of uterine rupture during pregnancy as one of the most serious complications, particularly after an extensive surgery (38). In a study of 23 women who underwent cytoreductive surgery, the myometrial thickness was measured by ultrasound or magnetic resonance imaging (73). The authors found a negative association between a residual myometrial thickness of <7 mm and unfavourable subsequent pregnancy outcomes, including miscarriages and two cases of uterine rupture in the first trimester. The authors concluded that the optimal wall thickness for preventing rupture after surgery might be within the range of 9 to 15 mm (73).

### 4.3 Non-surgical ablative techniques

Non-surgical ablative techniques have recently been developed to offer a non-invasive treatment alternative for adenomyosis-related infertility, particularly after conservative treatment has failed. Non-surgical ablation is based on three different technologies resulting in thermal ablation of the adenomyotic lesions: HIFU ablation, PMW ablation, and RF ablation (35). A recent review and meta-analysis comparing the fertility results of excisional vs. non-excisional techniques showed no statistically significant difference in the pooled estimates of pregnancy, miscarriage, and live birth rates between the two methods (74).

#### 4.3.1 HIFU ablation

Magnetic resonance imaging-guided or ultrasound-guided HIFU is probably the most thoroughly studied technology currently at clinicians’ disposal. HIFU ablation is based on the generation of ultrasound beams by an extra-corporeal transducer. These ultrasound waves can heat the target tissue through three mechanisms of action: thermally, cavitationally, and mechanically. After reaching a target temperature of >65°C, tissues undergo coagulation and necrosis. Appropriately targeting the ultrasound beam and checking for the expected heating effect in real time by magnetic resonance imaging or ultrasound substantially increase the safety and efficacy of HIFU ablation (75).

To date, HIFU ablation has mainly been developed and utilised in Asiatic regions, where it is quite prevalent. It appears to be very effective and safe in the treatment of symptomatic adenomyosis, especially in terms of alleviating dysmenorrhoea and abnormal uterine bleeding (76). There is no clear consensus regarding the indication for using HIFU ablation to improve fertility because clinical experience remains limited. However, it seems reasonable that HIFU ablation can positively impact fertility because ablation can be performed in a very targeted manner, reducing the risk of adverse effects on adjacent tissue such as necrosis and synechiae. Most importantly, the avoidance of uterine scarring may improve the safety of this treatment compared with surgical ablation of adenomyosis in cases of ongoing pregnancy (76). In a very recent systematic review and meta-analysis, reproductive outcomes after HIFU ablation of adenomyosis were evaluated in 10 studies involving 557 patients. The pooled estimated pregnancy rate was 53.4%, and the live birth rate was 35.2%. Nonetheless, the authors found substantial heterogeneity among the studies, limiting the strength of the results (77).

#### 4.3.2 RF ablation

RF is an alternative ablation technique involving US-guided implantation of an electrode in the lesion. A high-frequency alternating electrical current transmitted via RF generates heat by causing the molecules within cells to resonate, resulting in friction between them (78). Clinical experience with RF ablation of adenomyosis is less robust than that with HIFU ablation. A recent systematic review of seven studies indicated good efficacy in terms of reducing pain and abnormal uterine bleeding (79). Only two studies evaluated fertility outcomes after the treatment (80, 81). The larger of the two evaluated the outcomes of 74 infertile patients. Twenty-nine of the patients achieved 39 pregnancies, 84.6% of them ending with 24 live deliveries and 33.3% ending in spontaneous abortions. No case of uterine rupture occurred (80).

#### 4.3.3 PMW ablation

Ablation of adenomyosis by means of a PMW technique has recently been proposed and is the newest thermal ablation technique to be developed. A percutaneous electrode is inserted directly into the target tissue under ultrasound guidance. Coagulation necrosis of the lesion is induced by heating the tissues using electromagnetic energy, inducing polar water molecules to rapidly rotate and thus generate heat. Unfortunately, there is still very little clinical experience with this technology, particularly considering fertility (82).

## 5 Discussion

Adenomyosis remains a debilitating disease for women of reproductive age because it is associated with poor obstetrical outcomes and reduced fertility. In fact, increasing evidence suggests that adenomyosis adversely affects fertility outcomes. Several therapeutic options can be offered to these patients (Table 1). The therapeutic technique should be chosen according to the physician's expertise and the patient's clinical condition.

**Table 1:** Summary of evidence for pretreatment strategies on fertility outcomes in patients with adenomyosis.

Study		Year	Study design	Main results	Ref. nr.
GnRHa therapy					
Cao X., et al., The effectiveness of different down-regulating protocols on in vitro fertilization-embryo transfer in endometriosis: a meta-analysis		2020	Meta-analysis	GnRHa therapy improved the clinical pregnancy rates of patients with endometriosis	(50)
Wu, Y., et al., Long-term GnRH agonist pretreatment before frozen embryo transfer (FET) improves pregnancy outcomes in women with adenomyosis		2022	Comparative study GnRH agonist pretreatment during 3 month before FET. Three groups: (Group A) FET cycles following long-term GnRHa pretreatment; (Group B) fresh embryo transfer cycles with the ultra-long GnRHa protocol; (Group C) fresh embryo transfer cycles with the long GnRHa protocol	Significantly higher implantation and live birth rates for frozen embryo transfer following long-term GnRHa pretreatment	(10)
Aksenenko A.A., et al., Pre-treatment before *in vitro* fertilization and its effectiveness in patients with diffuse adenomyosis		2021	Comparative study Three groups: those who received treatment with GnRHa agents, combined oral contraceptives, or dienogest continuously for 3 months	GnRHa pretreatment increased the pregnancy rate by 5% to 12% per IVF attempt for women with stage 3 adenomyosis, whereas pretreatment with combined oral contraceptives and dienogest had no significant therapeutic effect	(51)
Niu, Z., et long-term pituitary downregulation before frozen embryo transfer could improve pregnancy outcomes in women with adenomyosis.al.		2013	Retrospective comparative study Two groups: combination of GnRHa with add-back therapy vs. add-back therapy only before frozen embryo transfer combination of GnRHa with add-back therapy vs. add-back therapy only before frozen embryo transfer	In down regulation + HRT group, clinical pregnancy, implantation and ongoing pregnancy rates were significantly higher than that of HRT group	(52)
Park, C.W., et al., Pregnancy rate in women with adenomyosis undergoing fresh or frozen embryo transfer cycles following gonadotropin-releasing hormone agonist treatment		2016	Comparative study Two groups: GnRHa pretreatment (for 2 or 3 month using 3.75 mg of goserelin) vs. no treatment before fresh embryo transfer	Frozen embryo transfer following GnRH agonist pretreatment tended to increase the pregnancy rate	(53)
LNG-IUS					
Liang, Z., et al., effect of pretreatment with a levonorgestrel-releasing intrauterine system on IVF and vitrified-warmed embryo transfer outcomes in women with adenomyosis		2019	Retrospective comparative study Two groups: women with adenomyosis undergoing IVF, LNG-IUS group vs control group (no therapy)	The ongoing pregnancy rate per transfer, the implantation rate and the clinical pregnancy rate were significantly higher in the LNG-IUS group than in the control group	(64)
Surgical ablative techniques					
Younes, G. and T. Tulandi, Conservative Surgery for Adenomyosis and Results: A Systematic Review.		2018	Meta-analysis	Surgical excision of adenomyosis is most probably effective for adenomyosis-related infertility	(67)
Saremi, A., et al., Treatment of adenomyomectomy in women with severe uterine adenomyosis using a novel technique		2014	Retrospective study Women who underwent laparoscopic adenomyomectomy	Of 70 patients who attempted pregnancy either naturally (*n* = 21) or using assisted reproduction technology (*n* = 49), 30% became pregnant, and 16 pregnancies reached full term	(68)
Kishi, Y., M. Yabuta, and F. Taniguchi, Who will benefit from uterus-sparing surgery in adenomyosis-associated subfertility?		2014	Retrospective study Women who underwent laparoscopic adenomyomectomy	Conservative surgery improved the fertility of women who had experienced IVF treatment failure, particularly those younger than 39 years of age	(69)
Huang, B.S., et al., Fertility outcome of infertile women with adenomyosis treated with the combination of a conservative microsurgical technique and GnRH agonist: long-term follow-up in a series of nine patients		2012	Retrospective study Women who underwent excision of adenomyosis followed by a 6-month course of GnRHa therapy	Higher clinical pregnancy and successful delivery rates in the surgery than medical therapy group	(70)
Wang, J.H., et al., Methotrexate therapy for cesarean section scar pregnancy with and without suction curettag		2009	Retrospective study Women treated with conservative surgery with or without GnRHa therapy	no differences in the reproductive outcomes between the two groups	(72)
Non-surgical ablative techniques					
HIFU					
Zhang, L., F. Rao, and R. Setzen, High intensity focused ultrasound for the treatment of adenomyosis: selection criteria, efficacy, safety and fertility		2017	Review article	Patients with adenomyosis who wished to conceive showed fovourable conception and live birth rates	(76)
Chen, Y., et al., Systematic review and meta-analysis of reproductive outcomes after high-intensity focused ultrasound (HIFU) treatment of adenomyosis		2024	Meta-analysis	There is evidence in favor HIFU to improve fertility in patients with adenomyosis, but is still very weak	(77)
RF ablation					
Nam, J.H., Pregnancy and symptomatic relief following ultrasound-guided transvaginal radiofrequency ablation in patients with adenomyosis		2020	Retrospective study Eighty-one patients who wanted to preserve their fertility and underwent RF ablation for adenomyosis	35.8% of the 81 patients achieved 39 pregnancies, 84.6% of them ending with 24 live deliveries	(80, 81)
PMW ablation					
Zhang, S., et al., Ultrasound-guided percutaneous microwave ablation of adenomyosis: a narrative review		2021	Narrative review	Almost no data on fertility	(82)

GnRHa, gonadotropin-releasing hormone; LNG-IUS, levonorgestrel-releasing intrauterine system; HIFU, high-intensity focused ultrasound; RF, radiofrequency; PMW, percutaneous microwave.

Pretreatment with either GnRHa therapy or LNG-IUS placement can reverse the patient's decreased fertility and improve the chances of pregnancy and successful IVF/ICSI cycles. The evidence supporting the use of GnRHa therapy is promising despite some studies showing contradictory results. A single study on the use of an LNG-IUS showed improvement in fertility-related outcomes. Further controlled trials are necessary to better demonstrate the relationship between pretreatment with GnRHa therapy or LNG-IUS placement and IVF/ICSI outcomes in women with adenomyosis.

Cytoreductive techniques appear to be a feasible option with high potential for positive effects on fertility. However, technical complexity limits the spread of these therapies in clinical practice, and their use is instead favoured in selected cases. Furthermore, the risk of major complications such as uterine rupture during pregnancy should be considered and discussed in advance with the patient. In this sense, thermal ablative techniques such as HIFU, RF, and PMW ablation may be effective alternative options. A recent review and meta-analysis comparing the fertility results of excisional vs. non-excisional techniques showed no statistically significant difference in the pooled estimates of pregnancy, miscarriage, and live birth rates between the two methods (74).

## 6 Conclusion

In conclusion, the management of adenomyosis-related infertility requires a tailored approach, considering the severity of the disease, patient preferences, and the available expertise. Both medical and surgical pretreatment strategies have shown potential in improving fertility outcomes, yet each comes with its own set of benefits and limitations. GnRHa and LNG-IUS are promising medical options, while surgical and non-surgical ablation techniques offer viable alternatives for those unresponsive to medical therapies. Continued research and well-designed clinical trials are essential to refine these strategies and develop robust, evidence-based guidelines for the treatment of adenomyosis-associated infertility.
